# Hepatocellular Carcinoma in Southeast Asian Americans: Epidemiologic Trends, Screening Challenges, and Policy Implications

**DOI:** 10.3390/healthcare14101314

**Published:** 2026-05-12

**Authors:** Ahauve M. Orusa, Abby M. Lohr, Khalid F. Abu-Zeinah, Irene G. Sia, Jennifer L. Ridgeway, Aminah Jatoi, Nguyen H. Tran

**Affiliations:** 1 Department of Medicine, Mayo Clinic, Rochester, MN 55905, USA; 2 Division of Epidemiology, Department of Quantitative Health Sciences, Mayo Clinic, Rochester, MN 55905, USA; 3 Division of Infectious Disease, Department of Medicine, Mayo Clinic, Rochester, MN 55905, USA; 4 Department of Oncology, Mayo Clinic, Rochester, MN 55905, USA

**Keywords:** hepatocellular carcinoma, Southeast Asian American, hepatitis B, screening

## Abstract

**Background**: Southeast Asian Americans (SEAAs) experience a disproportionately high burden of hepatocellular carcinoma (HCC), with incidence in several subgroups (i.e., Cambodian, Laotian, and Vietnamese individuals) reaching up to nine times that of non-Hispanic Whites. HCC in SEAAs is largely driven by chronic hepatitis B (HBV), hepatitis C (HCV), metabolic dysfunction–associated steatotic liver disease (MASLD), and alcohol-associated liver disease (ALD). Despite established screening guidelines, under-detection and delayed diagnosis remain common. **Objective**: To summarize epidemiologic patterns, risk factors, screening challenges, and potential interventions aimed at reducing HCC disparities among SEAAs. **Design and Methods**: This narrative review synthesized evidence from population based epidemiologic studies, community-based interventions, health services research, and policy analyses. Attention was given to studies reporting disaggregated SEAA subgroup data. Findings derived from SEAA specific studies were distinguished from evidence drawn from broader Asian American or general cirrhosis populations, with inferential steps explicitly noted where subgroup specific data were limited. **Key Findings**: HCC incidence varies widely across SEAA subgroups, with elevated HBV- and HCV-related HCC in Vietnamese, Cambodian, and Laotian communities, and increasing MASLD-related HCC including among lean individuals who fall outside many surveillance frameworks. Screening and surveillance remain suboptimal, with fewer than 30% of patients with cirrhosis receiving recommended semiannual HCC surveillance and even lower uptake among SEAAs. Barriers include low HBV/HCV screening rates, limited disease awareness, language barriers, underinsurance, provider knowledge gaps, and lack of automated EHR-based reminders. Structural challenges such as poverty, transportation barriers, and limited access to specialty care further delay diagnosis. **Proposed Interventions**: Culturally tailored outreach programs, bilingual navigators, and community-based screening initiatives have demonstrated improved HBV/HCV testing and linkage to care. AI-enabled EHR tools may enhance identification of high-risk patients, streamline follow-up, and increase surveillance adherence. Expanded use of non-invasive fibrosis assessment and recognition of MASLD-related risk in non-obese individuals may support earlier detection. Policy priorities include mandatory Asian subgroup data disaggregation, expanded insurance coverage, and strengthened community-level healthcare infrastructure. **Conclusions**: SEAAs face a substantial and preventable HCC burden. A coordinated approach combining culturally tailored community engagement, improved provider support systems, and policy reforms is essential to improving early detection and reducing HCC disparities in this diverse population.

## 1. Introduction

### Liver Cancer Burden Among Southeast Asian Americans

SEAAs are individuals in the U.S. who trace their ancestry to countries in Southeast Asia, including Myanmar (Burma), Thailand, Laos, Cambodia, Vietnam, Malaysia, Singapore, Indonesia, the Philippines, Brunei, and East Timor. In this review, these populations are grouped together for analytic purposes because of shared historical, migratory, and structural factors that shape liver cancer risk, particularly high endemic exposure to hepatitis B virus (HBV), refugee or immigrant status, language barriers, and limited access to culturally tailored preventive care. However, we explicitly recognize that hepatocellular carcinoma (HCC) risk, underlying etiologies, and screening patterns vary substantially across SEAA subgroups. Where available, this review prioritizes subgroup-specific evidence. In sections where data are limited to specific populations, particularly Vietnamese, Cambodian, and Laotian Americans, this is stated explicitly. In the absence of disaggregated subgroup data, broader Asian American or Asian Pacific Islander (API) -level evidence is discussed cautiously and interpreted as hypothesis-generating rather than definitive for all SEAA subgroups.

This diverse, growing population includes both immigrants and U.S.-born individuals of Southeast Asian (SEA) descent. SEAAs experience a disproportionately high burden of HCC, with incidence and mortality rates exceeding those of other racial and ethnic groups [1,2]. Unlike other racial and ethnic groups in the U.S., for whom heart disease remains the leading cause of death, cancer is the primary cause of mortality among Asian Americans, making HCC a critical concern [1,3].

HCC is the most common primary liver cancer, accounting for approximately 75–85% of liver cancer cases worldwide [4,5]. It typically arises in the setting of chronic liver disease and cirrhosis and is associated with high mortality due to delayed diagnosis and limited curative options [4,5].

HCC is primarily associated with chronic hepatitis B virus (HBV) and hepatitis C virus (HCV) infections, metabolic dysfunction–associated steatotic liver disease (MASLD), and alcohol-related liver disease (ALD) [6]. These diseases are largely preventable or modifiable, highlighting the potential for targeted interventions to reduce HCC incidence and mortality. War-related exposures such as dioxin may also contribute to HCC risk among SEAAs, but this remains understudied and overshadowed by viral and metabolic etiologies [7].

Among SEAA subgroups with available disaggregated data, particularly Vietnamese, Cambodian, and Laotian populations, HCC incidence varies substantially, ranging from 7 to 49 per 100,000 in men and 2–14 per 100,000 in women. Between 2003 and 2017, Vietnamese Americans experienced nearly triple the liver cancer mortality rate of non-Hispanic Whites. A 2025 California-based analysis (2010–2018) emphasized these disparities: Cambodian men and women had the highest HCV-related HCC incidence (15.5 and 6.3 per 100,000, respectively), followed by Vietnamese men and women (13.7 and 4.8 per 100,000, respectively) [6], compared with 4.8 and 1.1 per 100,000 in non-Latino White men and women, respectively [6]. Elevated HBV-related HCC incidence was also observed among SEAA subgroups, including Cambodians (18.3 for men, 3.4 for women) [6], compared with 0.3 and 0.0 per 100,000 in non-Latino whites, respectively [6]. Historical analyses show that Vietnamese, Cambodian, and Laotian Americans have experienced HCC incidence rates 8–9 times higher than non-Hispanic Whites, reflecting high chronic HBV prevalence among immigrants from these countries [8]. In contrast, HBV-related HCC is relatively rare among Japanese and SEAA populations, with rates comparable to those of White populations [6]. This may be due to differences in HBV genotypes, vaccination coverage, viral characteristics, and competing etiologies [9,10,11].

In analyses where subgroup-specific SEAA data are available, including a Florida-based study (2005–2018), HCC etiologies varied considerably by subgroup [12]. HBV-related HCC accounted for 38% of cases, followed by HCV (31.7%), MASLD (16.3%), and alcohol-related HCC (5%) [12]. MASLD-related HCC is defined as primary liver cancer that arises in the context of MASLD, a condition characterized by hepatic steatosis in individuals with metabolic risk factors such as obesity, type 2 diabetes, or dyslipidemia [13]. Predominating etiologies varied by subgroup: HBV predominated among SEAs (47.1%), MASLD among Filipinos (36.3%), and HCV among Vietnamese (42.9%). HBV and HCV were equally prevalent among South Asians. Sex differences were also observed, with HBV-related HCC most common among men and HCV-related HCC among women [12]. These subgroup-specific patterns should be interpreted cautiously when extrapolating to less studied SEAA populations.

Despite these findings, contemporary, granular data for SEAA subgroups appear limited outside of California and Florida, with few analyses extending beyond 2018. Given the substantial heterogeneity in HCC burden among SEAAs, subgroup-specific data are essential for developing culturally tailored prevention and screening strategies. Where possible, this paper includes disaggregated data to address gaps created by the lack of ethnic detail in federal surveys.

While the policy recommendations outlined in this review are intended to inform national strategies for reducing HCC disparities among SEAAs, their generalizability should be interpreted with caution. Much of the contemporary, disaggregated evidence informing these recommendations is derived from data collected primarily in California and Florida, states with large SEAA populations, distinct immigration histories, and relatively robust public health infrastructure. SEAA communities in other regions of the United States may differ substantially in healthcare access, insurance coverage, availability of culturally tailored services, and provider familiarity with HCC risk and surveillance guidelines. As a result, the feasibility and effectiveness of proposed interventions may vary across geographic contexts. National policy efforts should therefore incorporate mechanisms for regional adaptation and be accompanied by expanded investment in surveillance infrastructure and research in underrepresented states to support equitable implementation and impact nationwide (Table 1).

## 2. Methods

### 2.1. Study Design

This article was conducted as a narrative review to synthesize and contextualize existing evidence on HCC epidemiology, screening practices, and intervention strategies among SEAA populations. A narrative review approach was chosen to accommodate the heterogeneity of available data, which includes population-based epidemiologic studies, community-based interventions, health services research, and policy analyses. This methodology allows for integration of diverse study designs and evolving concepts that are not readily amenable to formal systematic review or meta-analytic techniques, particularly in the context of limited nationally representative data with detailed ethnic subgroup disaggregation.

### 2.2. Literature Search Strategy

A structured literature search was performed using PubMed/MEDLINE, Embase, and Google Scholar to identify relevant peer-reviewed publications. The search strategy incorporated a combination of controlled vocabulary terms, including Medical Subject Headings (MeSH), and free-text keywords related to HCC, hepatitis B and C, metabolic liver disease, screening, surveillance, Asian American populations, and Southeast Asian subgroups. Search terms were adapted to the indexing conventions of each database. In addition to electronic searches, reference lists of selected articles and relevant review papers were manually examined to identify additional pertinent studies. Given recognized limitations in national surveillance datasets, particular attention was paid to studies reporting disaggregated SEAA subgroup data, with geographic setting explicitly considered during evidence synthesis.

### 2.3. Study Selection

Study selection was guided by relevance to the objectives of the review. Titles and abstracts were initially screened to exclude articles that were clearly unrelated to HCC risk, screening, or intervention strategies in SEAA populations. Full-text review was subsequently performed for potentially relevant publications. Eligible studies included original research articles, population-based analyses, community-based intervention studies, review articles, and key policy or methodological papers addressing HCC or its major risk factors. Publications were limited to English-language articles and, where applicable, studies involving human subjects. Case reports with limited generalizability, conference abstracts without full manuscripts, and articles not directly aligned with the review focus were excluded unless they provided a uniquely informative context. Consistent with narrative review methodology, no formal methodological quality thresholds were applied, allowing inclusion of influential or hypothesis-generating studies where appropriate.

### 2.4. Data Extraction and Synthesis

Data were extracted qualitatively from included studies, with emphasis on study design, population characteristics, SEAA subgroup representation, geographic context, and key outcomes relevant to HCC risk, screening, and surveillance. Evidence was synthesized descriptively and thematically rather than quantitatively. Findings were contextualized across study designs and settings to identify recurring patterns, areas of agreement or divergence, and gaps in the existing literature. Quantitative estimates such as incidence rates or screening uptake were interpreted in relation to underlying data sources and regional scope and were not assumed to be nationally representative in the absence of supporting evidence. During evidence synthesis, findings were categorized according to whether they were derived from studies specific to SEAA subgroups, broader Asian American or API cohorts, or general cirrhosis populations. When conclusions draw on indirect evidence, the inferential step is stated explicitly to distinguish extrapolation from subgroup-specific data.

## 3. Epidemiology and Etiologic Patterns of HCC Among SEAAs

### 3.1. Risk Factors Contributing to HCC in Southeast Asian Americans

Evidence linking specific HCC risk factors to SEAA populations is uneven and geographically concentrated; therefore, this section distinguishes between findings supported by subgroup-specific data and broader patterns inferred from Asian American–level studies.

#### 3.1.1. Hepatitis B

HBV screening rates among high-risk Asian populations remain suboptimal, ranging from 30 to 50% among Asian immigrants, with fewer than 25% of those with chronic HBV being diagnosed [14]. Among SEAA subgroups with available community-based data, HBV prevalence rates are highest among individuals from Cambodia (11.9%), Vietnam (8.2%), and Laos (6.1%) [15,16]. National Health and Nutrition Examination Survey (NHANES) data estimates chronic HBV prevalence at 1.9% overall among non-Hispanic Asian adults (2017–2020) and 3.85% among foreign-born non-Hispanic Asian (2011–2016); however, NHANES underrepresents high-risk immigrants and does not reliably disaggregate Southeast Asian (SEA) subgroups, likely contributing to discrepancies with community studies [17,18,19]. In the absence of reliable national SEAA subgroup disaggregation, NHANES estimates likely underestimate HBV burden in high-risk SEAA populations. Furthermore, longitudinal data on HBV-associated HCC incidence among SEA subgroups remain limited due to inadequate ethnic disaggregation in public datasets.

From 2000 to 2015, U.S. age-adjusted HCC incidence rose from 5.56 to approximately 10.03 per 100,000 before declining to 9.20 per 100,000 by 2019 [20]. Disaggregated California data (1988–2012) show that SEA groups, specifically Vietnamese, Cambodian, and Laotian individuals, have strikingly elevated HCC risks, with incidence rates eight to nine times higher than non-Hispanic whites and more than twice as high as other Asian subgroups. Increases were particularly pronounced among Vietnamese and Laotian females [8].

This disproportionate burden is closely linked to high chronic HBV prevalence among these SEA communities, reaching 12.5% among Vietnamese and 13.6% among Laotians born outside the U.S. [8].

Despite elevated risk, SEAAs exhibit some of the lowest HCC screening rates. A San Francisco study of SEAAs with chronic HBV demonstrated declining HCC surveillance over time, from 67% in the first year after diagnosis to 24% by year ten [21]. This study reflects clinical practice patterns from the early 2000s to the late 2000s, and screening uptake may have evolved since that period with increased awareness, guideline updates, and public health interventions. However, contemporary longitudinal data describing HCC surveillance trajectories among SEAA subgroups remain limited.

#### 3.1.2. Hepatitis C

Chronic HCV infection significantly contributes to HCC risk; however, subgroup-specific data among SEAAs are limited and largely derived from select geographic regions. A Northern California cohort study reported HCV prevalence of 5.5% among Asian Americans, with Vietnamese (7.9%) exhibiting the highest rates [22]. Community-based screenings in the Baltimore–Washington region found HCV prevalence of 3.8% among Asian-born immigrants, with particularly high rates among individuals from Cambodia (10.8%) and Myanmar (4.9%) [16]. These findings are based primarily on community-based studies and may not extend uniformly across all SEAA subgroups.

#### 3.1.3. Metabolic Dysfunction-Associated Steatotic Liver Disease (MASLD)

As viral etiologies decline, metabolic and alcohol-related causes of HCC are increasing [23]. SEAA subgroup–specific data on MASLD-related HCC remain sparse; therefore, much of the available evidence is drawn from broader Asian American or API-level analyses. MASLD prevalence in the general U.S. population was estimated at 32.5% between 2017 and 2020 [24,25,26], but only approximately 18% among Asian Americans, though SEA subgroup-specific data are limited [25,27]. In California, MASLD-related HCC incidence was highest among API men (3.9 per 100,000) and women (1.8 per 100,000) [6], and was the leading cause of HCC among Filipino, Pacific Islander, and South Asian individuals, with rates exceeding those observed in White populations across all Asian subgroups [6]. MASLD-related HCC may develop in the absence of obesity and is influenced by genetic susceptibility, including PNPLA3 and TM6SF2 variants, and pro-oncogenic pathways involving inflammation and oxidative stress [28]. 

#### 3.1.4. Alcohol Associated Liver Disease (ALD)

ALD is another major contributor to HCC in the U.S. [6,29]. Among Asian American adults, alcohol use disorder prevalence increased from 3.5% (2015–2019) to 6.1% in recent years [6,30]. In California, ALD-related HCC incidence increased across SEA subgroups between 2010 and 2018, with projections suggesting ALD may become the leading cause of HCC-related mortality if current trends persist [30]. 

**Figure 1:**
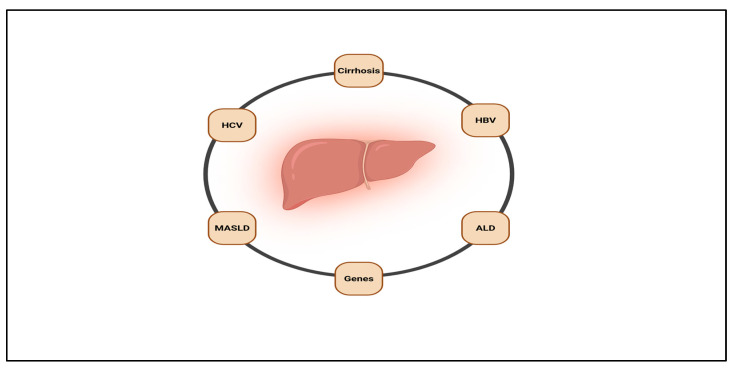
Major Drivers of HCC Development. Created in BioRender. Abu-Zeinah, K. (2026) https://BioRender.com/a5erduj (accessed on 15 February 2026).

Understanding the evolving etiologies of HCC is critical, but equally important is addressing barriers to effective screening and early detection among SEAA populations (Figure 1). 

## 4. Screening and Surveillance

### 4.1. Current Screening Recommendations

The U.S. Preventive Services Task Force (USPSTF) recommends one-time screening for HCV infection in all asymptomatic adults aged 18 to 79 years using anti-HCV antibody testing, with confirmatory HCV RNA testing for positive results [31]. Repeat testing is advised for individuals with ongoing risk factors, including injection drug use [31]. Screening is also recommended for pregnant individuals and those outside the standard age range who are at increased risk [31]. For HBV, USPSTF recommends screening asymptomatic, nonpregnant adolescents and adults at increased risk for HBV infection, including persons born in regions with high HBV prevalence, people who inject drugs, men who have sex with men, and household or sexual contacts of HBV-infected individuals [32]. The CDC now recommends universal HBV screening for all adults aged ≥ 18 years at least once in their lifetime using a triple panel test, and universal HCV screening for all adults and during each pregnancy [33,34,35]. Periodic testing is advised for individuals with ongoing risk factors for either infection [33,34,35].

For HCC, guidelines recommend surveillance every six months using abdominal ultrasound, with or without alpha-fetoprotein testing, for high-risk individuals [36]. This includes Asian men over age 40 and Asian women over age 50 with chronic HBV infection, individuals with cirrhosis of any etiology, those with perinatal HBV infection, and those with a family history of HCC [36].

### 4.2. Barriers, Challenges, and Additional Risk Factors for Screening and Surveillance

Evidence describing barriers to HCC screening and surveillance among SEAAs is derived from a heterogeneous body of literature, including studies specific to SEAA subgroups, broader Asian American cohorts, and general cirrhosis populations. Accordingly, this section distinguishes between barriers demonstrated directly in SEAA populations and those inferred from related groups that share structural, health system, or sociocultural characteristics.

Despite recommendations outlined in the previous section, surveillance among SEA remains suboptimal. Although many identified barriers are shared across multiple SEAA subgroups, much of the empirical evidence comes from studies conducted in Vietnamese, Cambodian, and Chinese American populations. Community-based studies in New York City report surveillance rates below 30% among SEA subgroups [37,38,39]. Nationally, fewer than 30% of patients with cirrhosis receive recommended semiannual HCC surveillance [40,41]. A Surveillance, Epidemiology, and End Results (SEER)-Medicare study found surveillance rates were highest among Asian patients (28.1%), compared to 12.2% for Black patients, 14.9% for White patients, and 16.8% for Hispanic patients [42]. However, these data are not disaggregated by Asian subgroups. Notably, while low surveillance rates have been consistently observed among patients with cirrhosis overall and in Asian American populations, the number of studies reporting SEAA-specific surveillance estimates remains limited. As such, interpretations regarding SEAAs necessarily integrate both subgroup-specific findings and extrapolation from closely related populations.

Emerging evidence suggests SEAAs with chronic HBV infection may develop HCC at younger ages, even in the absence of cirrhosis, and may acquire HCV before reaching standard screening thresholds, contributing to under-detection and delayed diagnosis [43]. Several barriers contribute to the low rates of screening and surveillance among SEAAs [44,45,46,47,48,49,50,51]. At the patient and provider level, both groups often lack sufficient awareness and knowledge regarding key risk factors for HCC, particularly chronic HBV infection [44,45,46,47,48,49,50,51]. 

Patient-level risk factors include demographic and socioeconomic factors such as age, sex, race/ethnicity, primary language, insurance status, and behavioral health risks, including tobacco, alcohol, and drug use [44,45,46,47,48,49,50,51]. Provider knowledge gaps have been documented primarily in studies of general cirrhosis populations and Asian American cohorts, and are extrapolated here as relevant to SEAA patients receiving care within similar healthcare settings [45,49,52]. Notably, screening uptake among Asian Americans with chronic HBV remains suboptimal [53]. A study found that 67% of Asian patients diagnosed with HCC had not undergone prior surveillance, often resulting in late-stage diagnosis and limited treatment options, while other studies reported that clinicians with lower HCC knowledge scores were more likely to perceive barriers to surveillance [37,41,50]. Additional provider-related obstacles include limited time during clinical visits, challenges in diagnosing cirrhosis, lack of automated surveillance systems, and lower adherence to surveillance guidelines among primary care providers compared to specialists [44,45,46,47,48,49,50,51].

In SEAA-specific and broader Asian American studies, language and communication barriers further compound these challenges: limited English proficiency is common among SEAA subgroups, particularly Vietnamese Americans, and impedes access to health information, navigation of insurance, and screening services [49,54]. Beyond this, socioeconomic and structural obstacles also hinder effective screening [47,55,56]. SEAAs disproportionately experience high poverty rates and are more likely to be uninsured or underinsured, making regular HCC screening less accessible [57]. Healthcare inequities, including restricted Medicaid access, inconsistent insurance acceptance, and under-resourced clinics, further curtail access to care [47,49,58,59,60]. Geographic disparities and limited provider availability also pose hurdles, and the rurality and associated travel burdens have been linked to poorer cancer outcomes, which is a pertinent concern for SEA communities who often face additional barriers related to transportation, employment, and childcare responsibilities [49,56,61]. 

While understanding these barriers is essential, identifying and implementing strategies to improve screening and surveillance is critical to reducing HCC disparities among SEAAs. 

## 5. Interventions to Reduce HCC Disparities

### 5.1. Community-Level Interventions

Most community-based interventions addressing viral hepatitis and liver cancer prevention have been conducted in selected Asian American or API populations, with limited direct evaluation in many SEAA subgroups. Reducing HCC incidence and mortality among SEAAs requires targeted research and tailored interventions developed in partnership with affected communities, with a particular focus on improving surveillance and screening. Within this framework, culturally tailored, community-based outreach programs have demonstrated success in mitigating barriers to preventive care among Asian American populations, including lack of insurance, cost concerns, and low perceived need for screening, by improving access to primary care and low-cost screening services [22,49,54,59,62,63,64,65,66]. The Jade Ribbon Campaign, launched by Stanford’s Asian Liver Center in 2001, utilized culturally resonant media, public events, and screening activities to raise HBV and liver cancer awareness among API communities [67]. Similarly, San Francisco Hep B Free, a citywide effort beginning in 2007, has offered free or low-cost testing and vaccination at community venues, positioning San Francisco as a national model for comprehensive API-targeted HBV interventions [68]. In Mississippi, community health worker–led efforts screened nearly 500 Vietnamese individuals for HBV and achieved high vaccination uptake [52]. Navigator-led programs in Philadelphia and New York City serving Chinese, Vietnamese, and Korean populations substantially improved follow-up rates (77% vs. 46% at six months; 91% vs. 61% at twelve months), highlighting the importance of bilingual, culturally concordant navigation [62]. Further, a liver cancer education intervention in the Baltimore–Washington region produced a more than fivefold increase in HBV screening among Chinese, Korean, and Vietnamese Americans [69]. A study based in metropolitan Atlanta emphasized the crucial importance of expanding insurance coverage and enhancing patient education as strategies to empower Vietnamese Americans to seek HBV testing and make informed health decisions [70]. These are just a few examples of successful community-based initiatives that can be modeled after for future interventions. While not all of these programs were designed exclusively for SEAAs, they provide structurally relevant models for addressing shared barriers. Despite these successes, most initiatives remain concentrated in coastal regions, and among the cited examples, the Baltimore–Washington intervention is one of the few that explicitly described the use of Community-Based Participatory Research (CBPR) principles in its design and implementation. Broader and more consistent incorporation of CBPR approaches may strengthen the cultural relevance, trust, and sustainability of future interventions.

### 5.2. Provider-Level Interventions

This section presents conceptual and proposed provider-level strategies for improving HCC surveillance using artificial intelligence (AI)–enabled electronic health record (EHR) tools. These approaches reflect extrapolation from broader hepatology and cancer screening literature rather than SEAA-specific implementation studies.

Integrating AI tools with EHRs offers a compelling strategy to improve provider adherence to HCC surveillance protocols. AI-enabled EHR systems can automatically identify patients eligible for surveillance based on risk factors such as chronic HBV/HCV, cirrhosis, MASLD, or country of birth, facilitating earlier detection [71,72,73]. These systems can prioritize SEA subpopulations by leveraging structured EHR fields like race, ethnicity, and preferred language; while specific to HCC, this mirrors broader AI-integration strategies in hepatology aimed at enhancing early detection and nuanced risk stratification [74,75,76]. Moreover, natural language processing can scan radiology reports for actionable phrases (e.g., “follow-up MRI suggested”) to automatically trigger best-practice advisories [77]. To operationalize follow-up, AI can generate automated tasks for providers, complete with one-click follow-up order sets and reminders if no action is taken within 72 h; simultaneously, check-in or sign-off interfaces can present default order templates like semiannual abdominal ultrasound with alpha-fetoprotein or MRI/CT when ultrasound is limited [78]. While there are other initiatives looking at using AI to improve hepatitis B surveillance and other diseases that disproportionately affect specific groups of people, these studies are in investigative or pilot phases and have not yet been integrated into routine clinical practice.

Patient engagement can be enhanced through automated reminders in preferred languages paired with culturally relevant education [79]. Leveraging frameworks proven effective in other cancer screening contexts, such as randomized EHR nudges that have successfully increased colorectal cancer (CRC) screening orders in primary care, offers a promising model [80,81]. These approaches are already being scaled for breast and population-based CRC screening and could be adapted effectively for HCC surveillance [80,81]. Crucially, once HBV is diagnosed, patients can be enrolled in HCC registries, enabling ongoing surveillance reminders and systematic follow-up [6,82]. Altogether, this integrated AI–EHR framework supports precision-focused implementation of HCC surveillance, enhancing timely detection and improving clinical outcomes. 

Another important aspect to consider at the provider level is the utilization of anti-viral therapy. HCV-related HCC incidence has declined in the US since 2014 and continued to do so, largely due to the widespread adoption of direct-acting antiviral (DAA) therapies. Studies indicate that DAA treatment has led to a significant reduction in the incidence of HCC among patients with HCV-related cirrhosis [83,84,85]. However, early occurrence and recurrence of HCC have been observed in some patients following DAA therapy, highlighting the need for ongoing surveillance [84].

### 5.3. Addressing MASLD-Related HCC 

Greater attention is needed for MASLD-related HCC, especially in non-obese and non-metabolic patients who often fall outside existing surveillance frameworks and are diagnosed at a later stage [6,27,86,87]. An estimated 15–46% of MASLD-associated HCC cases occur without cirrhosis, frequently in patients with advanced fibrosis, and these cases often present with larger, more advanced tumors [88]. Lean individuals with MASLD represent a particularly high-risk group, demonstrating higher liver-related mortality, increased insulin resistance, and adverse metabolic profiles despite normal body mass index [89,90]. 

In accordance with current guidance from major liver societies, including the American Association for the Study of Liver Diseases (AASLD) and the European Association for the Study of the Liver, routine HCC surveillance is recommended primarily for patients with cirrhosis, regardless of etiology, and is not universally advised for patients with MASLD without advanced fibrosis. However, both guidelines emphasize individualized, risk-based evaluation and heightened clinical vigilance in patients with multiple metabolic risk factors, evidence of advanced fibrosis, or persistently abnormal liver chemistries. Within this framework, clinicians should consider closer evaluation of lean individuals with MASLD who present with prediabetes, type 2 diabetes, or multiple cardiometabolic risk factors, and should treat incidental steatosis on imaging and unexplained elevations in aminotransferases as indicators warranting further assessment rather than reassurance. These recommendations are aligned with guideline-supported risk stratification principles but reflect application of expert clinical judgment in an area where prospective data and formal surveillance thresholds remain evolving [91,92].

## 6. Policy-Level Interventions

Policy interventions to reduce HCC disparities among SEAAs vary substantially in feasibility, resource requirements, and timeline for impact. Some strategies, such as improved data disaggregation and integration of risk-based screening prompts into existing health systems, are achievable within current administrative and regulatory frameworks. Other approaches, including large-scale community infrastructure investments, require sustained funding and cross-sector coordination. The following recommendations are therefore presented with attention to implementation feasibility, anticipated barriers, and relative priority.

Policy changes are essential to improve HCC screening and surveillance in SEAA populations [29]. Mandatory collection of disaggregated Asian American subgroup data is essential to accurately characterize HCC burden and screening disparities among SEAAs. At the state level, policies such as California’s AB-1726 demonstrate the feasibility of requiring health systems and public agencies to collect standardized subgroup identifiers across major diseases and demographic indicators. Extension of similar requirements to other high-SEAA states could be implemented through modifications to state cancer registries, Medicaid reporting requirements, and public health surveillance systems [93]. At the federal level, updating Office of Management and Budget racial and ethnic classification standards represents a more complex but high-impact strategy; intermediate steps could include incentivizing voluntary subgroup reporting within existing CDC and CMS datasets. Beyond improving epidemiologic precision, subgroup data disaggregation enables more equitable resource allocation, funding prioritization, and policy design by revealing disparities that are obscured by aggregate racial categories [6,94,95,96]. 

Key implementation barriers include variable institutional capacity for data system upgrades, concerns about respondent burden, and inconsistent use of standardized subgroup definitions across agencies, all of which will require coordinated technical guidance and funding support.

Addressing neighborhood-level disparities is equally crucial. SEAAs often live in low–socioeconomic status areas with limited healthcare infrastructure and higher rates of late-stage HCC diagnosis [55]. Healthcare access programs and community-level infrastructure investments such as bolstering Federally Qualified Health Centers, transportation support, and mobile clinics can help overcome geographic and structural barriers to early detection and care [97]. From a prioritization standpoint, policies that expand insurance coverage and reduce financial barriers represent the most immediately actionable levers for improving early HCC detection, while community infrastructure investments are likely to yield more gradual but durable gains in screening equity.

Finally, expansion of Medicaid and strict enforcement of no-cost preventive care provisions are vital components of this policy framework [98]. Insurance coverage consistently emerges as a determinant of stage at cancer diagnosis, with uninsured and underinsured populations more likely to present with late-stage disease across racial and ethnic groups, including SEAAs [98]. 

**Figure 2:**
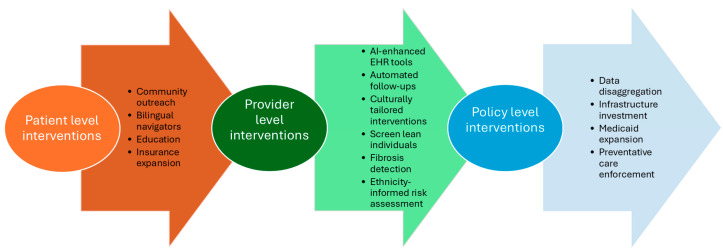
Proposed Strategies for Reducing HCC Burden.

Taken together, these policy strategies highlight the importance of aligning feasibility with impact: prioritizing policy actions that are achievable within existing health systems in the near term, while concurrently building the data and community infrastructure necessary to support more tailored and equitable HCC prevention efforts over time (Figure 2).

## 7. Discussion

### Interpretation of Findings and Evidence Gaps

This review should be interpreted in light of several important limitations inherent to the available evidence base. As a narrative review, this work does not include formal quality assessment or quantitative synthesis, and its conclusions rely on descriptive and thematic integration of heterogeneous study designs. Although a structured and transparent search strategy was used, the findings are constrained by the characteristics of the published literature, including the geographic concentration of detailed SEAA subgroup data in a limited number of regions, most notably California and Florida. As a result, estimates of HCC incidence, screening practices, and intervention effectiveness may not be generalizable to SEAA communities in other parts of the United States, where immigration histories, healthcare infrastructure, insurance coverage, and access to culturally tailored services differ substantially. In several areas, conclusions necessarily draw on evidence from broader Asian American or general cirrhosis populations due to persistent gaps in SEAA subgroup–specific data. These inferential steps have been explicitly noted to avoid overgeneralization. Together, these limitations underscore the need for cautious interpretation of national implications and highlight the critical importance of expanded data disaggregation and geographically diverse research to more accurately characterize HCC risk and screening disparities among SEAA populations.

## 8. Conclusions

SEAAs face a disproportionately high burden of HCC, driven by complex and interrelated factors including chronic viral hepatitis, MASLD, and ALD. Despite established screening and surveillance guidelines, adherence remains suboptimal due to multifaceted barriers at the patient, provider, and system levels.

Culturally tailored interventions that are co-created with the communities most impacted, such as community-based outreach programs and bilingual patient navigators, have shown promise in improving screening uptake and continuity of care. Additionally, while more research is needed regarding how AI can be used to improve screening uptake for diseases such as HBV, AI-enhanced EHR systems should be further explored. Expanding these models and integrating them into routine practice can help close gaps in early detection and treatment. 

MASLD-related HCC, particularly in lean individuals, presents a growing challenge that requires further research and clinical attention. Providers must adopt a proactive approach to screening, incorporating non-invasive tools and considering ethnicity-specific risk factors even when not explicitly addressed in current guidelines. 

At the policy level, improving access to care, expanding insurance coverage, and investing in community infrastructure are critical. Most importantly, disaggregating Asian American data is essential to reveal subgroup-specific disparities, accurately estimate HCC burden, and inform culturally and clinically tailored interventions. A coordinated, multi-level strategy rooted in equity and community partnership is essential to reducing HCC disparities among SEAA communities. Importantly, translating these policy recommendations into practice will require deliberate prioritization, sustained funding, and coordination across federal, state, and local stakeholders, particularly in regions with emerging SEAA populations.

## Figures and Tables

**Table 1:** Incidence of Hepatocellular Carcinoma and Associated Etiologies Among Southeast Asian American Subgroups.

HCC Incidence Per 100,000 Population					
	Total	HCV	MASLD	ALD	HBV
Filipino men	13.7 (12.8–14.8)	1.7 (1.4–2.1)	6.2 (5.5–6.9)	1.2 (0.9–1.5)	3.6 (3.2–4.1)
South Asian men	8.4 (7.2–9.8)	2.5 (1.9–3.2)	3.5 (2.6–4.5)	1.0 (0.6–1.4)	1.1 (0.7–1.6)
Vietnamese men	40.1 (37.9–42.5)	13.7 (12.4–15.1)	5.8 (4.9–6.8)	0.9 (0.6–1.3)	17.4 (16.0–19.0)
Cambodian men	48.6 (39.4–59.0)	15.5 (10.1–22.4)	NA	NA	18.3 (13.6–24.0)
Hmong men	20.8 (13.8–29.7)	NA	NA	NA	10.0 (5.7–16.1)
Laotian men	46.1 (37.3–56.3)	8.6 (5.0–13.6)	NA	NA	25.0 (18.9–32.5)
Thai men	33.2 (22.0–47.7)	NA	NA	NA	14.8 (8.7–23.8)
Non-Latino White men	9.2 (9.1–9.4)	4.8 (4.7–4.9)	2.1 (2.1–2.2)	1.4 (1.3–1.4)	0.3 (0.2–0.3)
Filipino women	4.4 (4.0–4.9)	0.6 (0.4–0.8)	2.5 (2.2–2.9)	NA	0.8 (0.6–1.0)
South Asian women	2.4 (1.8–3.2)	1.0 (0.7–1.5)	1.1 (0.7–1.7)	NA	0.2 (0.0–0.4)
Vietnamese women	11.1 (10.0–12.4)	4.8 (4.0–5.7)	2.1 (1.6–2.7)	NA	3.5 (2.9–4.2)
Cambodian women	14.0 (10.6–18.2)	6.3 (4.1–9.2)	NA	NA	3.4 (1.9–5.6)
Hmong women	NA	NA	NA	NA	NA
Laotian women	9.6 (6.0–14.6)	NA	NA	NA	5.4 (2.7–9.5)
Thai women	7.2 (4.4–11.5)	NA	NA	NA	2.7 (1.2–5.8)
Non-Latino White women	2.6 (2.5–2.7)	1.1 (1.1–1.2)	0.8 (0.8–0.9)	0.2 (0.2–0.3)	0.0 (0.0–0.1)

Abbreviations: HCC, hepatocellular carcinoma; HCV, hepatitis C virus; MASLD, metabolic dysfunction steatotic liver disease; ALD, alcohol-associated liver disease; HBV, hepatitis B virus. Adapted from: Pinheiro PS et al. *JAMA Netw Open.* 2025; 8(3): e252208. PMID: 40146106 [6]. Data availability varies by subgroup; absence of data does not imply absence of risk.

## Data Availability

This study did not involve collection of primary data. All sources used in the review are publicly available in the published literature and are cited within the manuscript. No additional datasets were generated or analyzed.

## References

[1] Singal A.G., Murphy C.C. (2019). Hepatocellular Carcinoma: A Roadmap to Reduce Incidence and Future Burden. J. Natl. Cancer Inst..

[2] Yang B., Liu J.B., So S.K., Han S.S., Wang S.S., Hertz A., Shariff-Marco S., Lin Gomez S., Rosenberg P.S., Nguyen M.H. (2018). Disparities in hepatocellular carcinoma incidence by race/ethnicity and geographic area in California: Implications for prevention. Cancer.

[3] Lee R.J., Madan R.A., Kim J., Posadas E.M., Yu E.Y. (2021). Disparities in Cancer Care and the Asian American Population. Oncologist.

[4] El-Serag H.B. (2002). Hepatocellular carcinoma: An epidemiologic view. J. Clin. Gastroenterol..

[5] Llovet J.M., Kelley R.K., Villanueva A., Singal A.G., Pikarsky E., Roayaie S., Lencioni R., Koike K., Zucman-Rossi J., Finn R.S. (2021). Hepatocellular carcinoma. Nat. Rev. Dis. Primers.

[6] Pinheiro P.S., Zhang J., Setiawan V.W., Cranford H.M., Wong R.J., Liu L. (2025). Liver Cancer Etiology in Asian Subgroups and American Indian, Black, Latino, and White Populations. JAMA Netw. Open.

[7] Wiberg K., Aberg A., McKone T.E., Tysklind M., Hanberg A., MacLeod M. (2007). Model selection and evaluation for risk assessment of dioxin-contaminated sites. Ambio.

[8] Pham C., Fong T.L., Zhang J., Liu L. (2018). Striking Racial/Ethnic Disparities in Liver Cancer Incidence Rates and Temporal Trends in California, 1988–2012. J. Natl. Cancer Inst..

[9] Lim R.Y., Koh B., Ng C.H., Kulkarni A.V., Liu K., Wijarnpreecha K., Kim B.K., Muthiah M.D., Lee S.W., Zheng M.H. (2025). Hepatocellular Carcinoma Surveillance and Survival in a Contemporary Asia-Pacific Cohort. JAMA Netw. Open.

[10] Chan H.L., Hui A.Y., Wong M.L., Tse A.M., Hung L.C., Wong V.W., Sung J.J. (2004). Genotype C hepatitis B virus infection is associated with an increased risk of hepatocellular carcinoma. Gut.

[11] Lin C.-L., Kao J.-H. (2021). Prevention of hepatitis B virus-related hepatocellular carcinoma. Hepatoma Res..

[12] Cranford H.M., Jones P.D., Wong R.J., Liu Q., Kobetz E.N., Reis I.M., Koru-Sengul T., Pinheiro P.S. (2024). Hepatocellular Carcinoma Etiology Drives Survival Outcomes: A Population-Based Analysis. Cancer Epidemiol. Biomark. Prev..

[13] Huang D.Q., El-Serag H.B., Loomba R. (2021). Global epidemiology of NAFLD-related HCC: Trends, predictions, risk factors and prevention. Nat. Rev. Gastroenterol. Hepatol..

[14] Win A., King S., Wu G., Kwon S. (2023). Hepatitis B virus screening in Asian immigrants: Community-based campaign to increase screening and linkage to care: A cross-sectional study. Health Sci. Rep..

[15] Toy M., Wei B., Virdi T.S., Le A., Trinh H., Li J., Zhang J., Hsing A.W., So S.K., Nguyen M.H. (2018). Racial/ethnic- and county-specific prevalence of chronic hepatitis B and its burden in California. Hepatol. Med. Policy.

[16] Juon H.S., Ha E., Kim F., Trang A., Pan J., Blanchard J. (2019). Prevalence of Viral Hepatitis in Foreign-Born Populations in the Baltimore-Washington Metropolitan Area, 2009–2015. J. Community Health.

[17] Bixler D., Barker L., Lewis K., Peretz L., Teshale E. (2023). Prevalence and awareness of Hepatitis B virus infection in the United States: January 2017–March 2020. Hepatol. Commun..

[18] Le M.H., Yeo Y.H., Cheung R., Henry L., Lok A.S., Nguyen M.H. (2020). Chronic Hepatitis B Prevalence Among Foreign-Born and U.S.-Born Adults in the United States, 1999–2016. Hepatology.

[19] Conners E.E., Panagiotakopoulos L., Hofmeister M.G., Spradling P.R., Hagan L.M., Harris A.M., Rogers-Brown J.S., Wester C., Nelson N.P. (2023). Screening and Testing for Hepatitis B Virus Infection: CDC Recommendations—United States, 2023. Morb. Mortal. Wkly. Rep. Recomm. Rep..

[20] O’Brien T.R., Devesa S.S., Koshiol J., Marrero J.A., Shiels M.S. (2023). Decreasing incidence of hepatocellular carcinoma among most racial groups: SEER-22, 2000–2019. Cancer Med..

[21] Sarkar M., Stewart S., Yu A., Chen M.S., Nguyen T.T., Khalili M. (2012). Hepatocellular carcinoma screening practices and impact on survival among hepatitis B-infected Asian Americans. J. Viral Hepat..

[22] Lin O.N., Chang C., Lee J., Do A., Martin M., Martin A., Nguyen M.H. (2017). HCV Prevalence in Asian Americans in California. J. Immigr. Minor. Health.

[23] Ma Y., Wang J., Xiao W., Fan X. (2024). A review of MASLD-related hepatocellular carcinoma: Progress in pathogenesis, early detection, and therapeutic interventions. Front. Med..

[24] Younossi Z.M., de Avila L., Racila A., Nader F., Paik J., Henry L., Stepanova M. (2025). Prevalence and predictors of cirrhosis and portal hypertension in the United States. Hepatology.

[25] Paik J.M., Hobbs K., Gupta A., Alkalbani R.J., Reyes M.A., Younossi Z.M. (2025). Prevalence of MASLD, Met-ALD, and ALD and Associated Fibrosis Among US Adults: Insights from NHANES 2017 to 2023. J. Clin. Gastroenterol..

[26] Lee B.P., Dodge J.L., Terrault N.A. (2024). National prevalence estimates for steatotic liver disease and subclassifications using consensus nomenclature. Hepatology.

[27] Unalp-Arida A., Ruhl C.E. (2025). Prevalence of metabolic dysfunction-associated steatotic liver disease and fibrosis defined by liver elastography in the United States using National Health and Nutrition Examination Survey 2017-March 2020 and August 2021-August 2023 data. Hepatology.

[28] Aung T.C.Z., Boonkaew B., Chayanupatkul M., Poovorawan K., Chuaypen N., Tangkijvanich P. (2025). The Distribution and Survival Association of Genetic Polymorphisms in Thai Patients with Hepatocellular Carcinoma According to Underlying Liver Disease. Genes.

[29] Danpanichkul P., Duangsonk K., Kalligeros M., Fallon M.B., Vuthithammee C., Pan C.W., Saokhieo P., Derrick W., Pang Y., Chen V.L. (2025). Alcohol-Related Liver Disease, Followed by Metabolic Dysfunction-Associated Steatotic Liver Disease, Emerges as the Fastest-Growing Aetiologies for Primary Liver Cancer in the United States. Aliment. Pharmacol. Ther..

[30] Pinheiro P.S., Jones P.D., Medina H., Cranford H.M., Koru-Sengul T., Bungum T., Wong R., Kobetz E.N., McGlynn K.A. (2024). Incidence of Etiology-specific Hepatocellular Carcinoma: Diverging Trends and Significant Heterogeneity by Race and Ethnicity. Clin. Gastroenterol. Hepatol..

[31] U.S. Preventive Services Task Force Hepatitis C Virus Infection in Adolescents and Adults: Screening. https://www.uspreventiveservicestaskforce.org/uspstf/recommendation/hepatitis-c-screening.

[32] U.S. Preventive Services Task Force Hepatitis B Virus Infection in Adolescents and Adults: Screening. https://www.uspreventiveservicestaskforce.org/uspstf/recommendation/hepatitis-b-virus-infection-screening.

[33] Clinical Testing and Diagnosis for Hepatitis B. https://www.cdc.gov/hepatitis-b/hcp/diagnosis-testing/index.html.

[34] Significant Update to Hepatitis B Screening and Testing Recommendations. https://www.cdc.gov/nchhstp/director-letters/updated-hepatitis-b-screening-recommendations.html.

[35] Clinical Screening and Diagnosis for Hepatitis C. https://www.cdc.gov/hepatitis-c/hcp/diagnosis-testing/index.html.

[36] Frenette C.T., Isaacson A.J., Bargellini I., Saab S., Singal A.G. (2019). A Practical Guideline for Hepatocellular Carcinoma Screening in Patients at Risk. Mayo Clin. Proc. Innov. Qual. Outcomes.

[37] Wong R.J., Jones P.D., Niu B., Pinheiro P., Thamer M., Kshirsagar O., Zhang Y., Fass R., Therapondos G., Singal A.G. (2025). Hepatocellular Carcinoma Surveillance in Patients with Cirrhosis at US Safety-Net Health Systems. Clin. Transl. Gastroenterol..

[38] Van Manh A.L., Blondeau-Lecomte E., Makoni N., Jandorf L., Perumalswami P. (2020). Identifying Factors Associated with Cancer Screening in Immigrant Populations Living in New York City. J. Community Health.

[39] Pollack H.J., Kwon S.C., Wang S.H., Wyatt L.C., Trinh-Shevrin C., Asian American Hepatitis B Program Coalition (2014). Chronic hepatitis B and liver cancer risks among Asian immigrants in New York City: Results from a large, community-based screening, evaluation, and treatment program. Cancer Epidemiol. Biomark. Prev..

[40] Wolf E., Rich N.E., Marrero J.A., Parikh N.D., Singal A.G. (2021). Use of Hepatocellular Carcinoma Surveillance in Patients with Cirrhosis: A Systematic Review and Meta-Analysis. Hepatology.

[41] Wong R.J., Jones P.D., Niu B., Therapondos G., Thamer M., Kshirsagar O., Zhang Y., Pinheiro P., Kyalwazi B., Fass R. (2024). Clinician-Level Knowledge and Barriers to Hepatocellular Carcinoma Surveillance. JAMA Netw. Open.

[42] Davila J.A., Morgan R.O., Richardson P.A., Du X.L., McGlynn K.A., El-Serag H.B. (2010). Use of surveillance for hepatocellular carcinoma among patients with cirrhosis in the United States. Hepatology.

[43] Wan D.W., Tzimas D., Smith J.A., Kim S., Araujo J., David R., Lobach I., Sarpel U. (2011). Risk factors for early-onset and late-onset hepatocellular carcinoma in Asian immigrants with hepatitis B in the United States. Am. J. Gastroenterol..

[44] Beal E.W., McNamara M., Owen M., McAlearney A.S., Tsung A. (2024). Interventions to Improve Surveillance for Hepatocellular Carcinoma in High-Risk Patients: A Scoping Review. J. Gastrointest. Cancer.

[45] Dalton-Fitzgerald E., Tiro J., Kandunoori P., Halm E.A., Yopp A., Singal A.G. (2015). Practice patterns and attitudes of primary care providers and barriers to surveillance of hepatocellular carcinoma in patients with cirrhosis. Clin. Gastroenterol. Hepatol..

[46] Feng G.-H., Yue Q.-Q., Zhao K.-H., Peng T., Tang T., Sun Y.-X., Meng X.-R., Huang L.-L., Zeng X., Zeng Y. (2024). Factors affecting the compliance of hepatocellular carcinoma screening among high-risk populations: A systematic review and meta-analysis. Public Health Nurs..

[47] Kronenfeld J.P., Goel N. (2021). An Analysis of Individual and Contextual-Level Disparities in Screening, Treatment, and Outcomes for Hepatocellular Carcinoma. J. Hepatocell. Carcinoma.

[48] Li C., Lu X., Xiao J., Chan C.W.H. (2022). ‘We can bear it!’ Unpacking barriers to hepatocellular carcinoma screening among patients with hepatitis B: A qualitative study. J. Clin. Nurs..

[49] Liu D., Schuchard H., Burston B., Yamashita T., Albert S. (2021). Interventions to Reduce Healthcare Disparities in Cancer Screening Among Minority Adults: A Systematic Review. J. Racial Ethn. Health Disparities.

[50] Parikh N.D., Tayob N., Al-Jarrah T., Kramer J., Melcher J., Smith D., Marquardt P., Liu P.H., Tang R., Kanwal F. (2022). Barriers to Surveillance for Hepatocellular Carcinoma in a Multicenter Cohort. JAMA Netw. Open.

[51] Simmons O.L., Feng Y., Parikh N.D., Singal A.G. (2019). Primary Care Provider Practice Patterns and Barriers to Hepatocellular Carcinoma Surveillance. Clin. Gastroenterol. Hepatol..

[52] Funchess T.T., Fastring D., Walker V., Sutton V.D., Nguyen C., Le D., Tran X., Nguyen K., Nguyen J. (2022). Hepatitis B Screening, Vaccination, and Linkage to Care: Lessons Learned from a Mississippi Vietnamese Community. Prog. Community Health Partnersh..

[53] Ma G.X., Tan Y., Wang M.Q., Yuan Y., Chae W.G. (2010). Hepatitis B screening compliance and non-compliance among Chinese, Koreans, Vietnamese and Cambodians. Clin. Med. Insights Gastroenterol..

[54] Zovich B., Block S.J., Borondy-Jenkins F., Chen T., Moraras K., Afoakwah J., Dong M., Cohen C. (2024). The Role of Culturally Appropriate Mediated Communication Strategies to Reduce Hepatitis B and Liver Cancer Disparities. J. Health Commun..

[55] Sangaramoorthy M., Yang J., Guan A., DeRouen M.C., Tana M.M., Somsouk M., Thompson C.A., Gibbons J., Ho C., Chu J.N. (2022). Asian American/Pacific Islander and Hispanic Ethnic Enclaves, Neighborhood Socioeconomic Status, and Hepatocellular Carcinoma Incidence in California: An Update. Cancer Epidemiol. Biomark. Prev..

[56] Sempokuya T., Pan C.-W., Pattison R.J., Choi C., Nogimura A., Wong L.L. (2023). Disparities in Hepatocellular Carcinoma Outcomes Among Subgroups of Asians and Pacific Islanders: A SEER Database Study. J. Immigr. Minor. Health.

[57] Beydoun H.A., Tsai J. (2024). Screening rates for hepatitis B and C among low-income US veterans: Data from the National Veteran Homeless and Other Poverty Experiences Study. J. Viral Hepat..

[58] Ladhani S., Ohri A., Wong R.J. (2020). Disparities in Hepatocellular Carcinoma Surveillance: Dissecting the Roles of Patient, Provider, and Health System Factors. J. Clin. Gastroenterol..

[59] Vu M., Huynh V.N., Berg C.J., Allen C.G., Nguyen P.-L.H., Tran N.-A., Srivanjarean Y., Escoffery C. (2021). Hepatitis B Testing Among Vietnamese in Metropolitan Atlanta: The Role of Healthcare-Related and Acculturation-Related Factors. J. Community Health.

[60] Wu G., Augustine N.T., Kwon S.S. (2022). Preventive Cancer Screening in Asian Americans: Need for Community Outreach Programs. Asian Pac. J. Cancer Prev..

[61] Li D.Y., VoPham T., Tang M.C., Li C.I. (2022). Disparities in Risk of Advanced-Stage Liver Cancer and Mortality by Race and Ethnicity. J. Natl. Cancer Inst..

[62] Ma G.X., Zhu L., Tan Y., Zhai S., Ma X., Ogunwobi O.O., Yang W.J., Ting T., Kim S., Wang M.Q. (2023). A Comparative Trial of Improving Care for Underserved Asian Americans Infected with Hepatitis B Virus. Dig. Dis. Sci..

[63] Chen M.S., Chow E.A., Nguyen T.T. (2018). The Asian American Network for Cancer Awareness, Research, and Training (AANCART)’s contributions toward reducing Asian American cancer health disparities, 2000–2017. Cancer.

[64] Chen T., Borondy-Jenkins F., Zovich B., Block S.J., Moraras K., Chan A., Cohen C. (2024). Existing knowledge, myths, and perceptions about hepatitis B and liver cancer within highly impacted immigrant communities. J. Virus Erad..

[65] Escriba-Aguir V., Rodriguez-Gomez M., Ruiz-Perez I. (2016). Effectiveness of patient-targeted interventions to promote cancer screening among ethnic minorities: A systematic review. Cancer Epidemiol..

[66] Hong Y.A., Yee S., Bagchi P., Juon H.-S., Kim S.C., Le D. (2022). Social media-based intervention to promote HBV screening and liver cancer prevention among Korean Americans: Results of a pilot study. Digit. Health.

[67] Chao S.D., Chang E.T., Le P.V., Prapong W., Kiernan M., So S.K. (2009). The Jade Ribbon Campaign: A model program for community outreach and education to prevent liver cancer in Asian Americans. J. Immigr. Minor. Health.

[68] Bailey M.B., Shiau R., Zola J., Fernyak S.E., Fang T., So S.K., Chang E.T. (2011). San Francisco hep B free: A grassroots community coalition to prevent hepatitis B and liver cancer. J. Community Health.

[69] Juon H.S., Park B.J. (2013). Effectiveness of a culturally integrated liver cancer education in improving HBV knowledge among Asian Americans. Prev. Med..

[70] Frew P.M., Alhanti B., Vo-Green L., Zhang S., Liu C., Nguyen T., Schamel J., Saint-Victor D.S., Nguyen M.L. (2014). Multilevel factors influencing hepatitis B screening and vaccination among Vietnamese Americans in Atlanta, Georgia. Yale J. Biol. Med..

[71] Romeo M., Dallio M., Napolitano C., Basile C., Di Nardo F., Vaia P., Iodice P., Federico A. (2025). Clinical Applications of Artificial Intelligence (AI) in Human Cancer: Is It Time to Update the Diagnostic and Predictive Models in Managing Hepatocellular Carcinoma (HCC)?. Diagnostics.

[72] Sahoo P., Kundu M., Begum J. (2025). Artificial Intelligence in Cancer Diagnosis: A Game-changer in Healthcare. Curr. Pharm. Biotechnol..

[73] Thaker N.G., Dicker A.P., Loaiza-Bonilla A., Wallace A., Kolman D., Godshalk Ruggles A., Manda S., Frank S.J., Orio P., Doria C. (2024). The Role of Artificial Intelligence in Early Cancer Detection: Exploring Early Clinical Applications. AI Precis. Oncol..

[74] Martinino A., Aloulou M., Chatterjee S., Scarano Pereira J.P., Singhal S., Patel T., Kirchgesner T.P., Agnes S., Annunziata S., Treglia G. (2022). Artificial Intelligence in the Diagnosis of Hepatocellular Carcinoma: A Systematic Review. J. Clin. Med..

[75] Mohsen F., Ali H., El Hajj N., Shah Z. (2022). Artificial intelligence-based methods for fusion of electronic health records and imaging data. Sci. Rep..

[76] Gichoya J.W., Banerjee I., Bhimireddy A.R., Burns J.L., Celi L.A., Chen L.C., Correa R., Dullerud N., Ghassemi M., Huang S.C. (2022). AI recognition of patient race in medical imaging: A modelling study. Lancet Digit. Health.

[77] Liu H., Xu Y., Zhang Z., Wang N., Huang Y., Hu Y., Yang Z., Jiang R., Chen H. (2020). A Natural Language Processing Pipeline of Chinese Free-text Radiology Reports for Liver Cancer Diagnosis. IEEE Access.

[78] Calderaro J., Seraphin T.P., Luedde T., Simon T.G. (2022). Artificial intelligence for the prevention and clinical management of hepatocellular carcinoma. J. Hepatol..

[79] Kann B.H., Hosny A., Aerts H. (2021). Artificial intelligence for clinical oncology. Cancer Cell.

[80] Patel M.S., Volpp K.G., Small D.S., Wynn C., Zhu J., Yang L., Honeywell S., Day S.C. (2016). Using active choice within the electronic health record to increase physician ordering and patient completion of high-value cancer screening tests. Healthcare.

[81] Elfakki F.A.M., Alshammari K.I., Aljamani M.Y., Alshammari W.I. (2024). A nudge strategy to increase the uptake of colorectal cancer screening in Saudi Arabia: A pragmatic randomized trial in the Hail region. J. Fam. Med. Prim. Care.

[82] Yom S., Lor M. (2022). Advancing Health Disparities Research: The Need to Include Asian American Subgroup Populations. J. Racial Ethn. Health Disparities.

[83] Waziry R., Hajarizadeh B., Grebely J., Amin J., Law M., Danta M., George J., Dore G.J. (2017). Hepatocellular carcinoma risk following direct-acting antiviral HCV therapy: A systematic review, meta-analyses, and meta-regression. J. Hepatol..

[84] Calvaruso V., Cabibbo G., Cacciola I., Petta S., Madonia S., Bellia A., Tine F., Distefano M., Licata A., Giannitrapani L. (2018). Incidence of Hepatocellular Carcinoma in Patients With HCV-Associated Cirrhosis Treated with Direct-Acting Antiviral Agents. Gastroenterology.

[85] Kilany S., Ata L., Gomaa A., Sabry A., Nada A., Tharwa E.S., Badra G., Abogabal A., Elwaraky M., Moaz E. (2021). Decreased Incidence of Hepatocellular Carcinoma after Directly Acting Antiviral Therapy in Patients with Hepatitis C-Related Advanced Fibrosis and Cirrhosis. J. Hepatocell. Carcinoma.

[86] Shen T.H., Wu C.H., Lee Y.W., Chang C.C. (2024). Prevalence, trends, and characteristics of metabolic dysfunction-associated steatotic liver disease among the US population aged 12–79 years. Eur. J. Gastroenterol. Hepatol..

[87] Tan D.J.H., Tamaki N., Kim B.K., Wijarnpreecha K., Aboona M.B., Faulkner C., Kench C., Salimi S., Sabih A.H., Lim W.H. (2025). Prevalence of Low FIB-4 in MASLD-Related Hepatocellular Carcinoma: A Multicentre Study. Aliment. Pharmacol. Ther..

[88] Piscaglia F., Svegliati-Baroni G., Barchetti A., Pecorelli A., Marinelli S., Tiribelli C., Bellentani S., HCC-NAFLD Italian Study Group (2016). Clinical patterns of hepatocellular carcinoma in nonalcoholic fatty liver disease: A multicenter prospective study. Hepatology.

[89] Ha J., Yim S.Y., Karagozian R. (2023). Mortality and Liver-Related Events in Lean Versus Non-Lean Nonalcoholic Fatty Liver Disease: A Systematic Review and Meta-analysis. Clin. Gastroenterol. Hepatol..

[90] Han S.K., Baik S.K., Kim M.Y. (2023). Non-alcoholic fatty liver disease: Definition and subtypes. Clin. Mol. Hepatol..

[91] Rinella M.E., Neuschwander-Tetri B.A., Siddiqui M.S., Abdelmalek M.F., Caldwell S., Barb D., Kleiner D.E., Loomba R. (2023). AASLD Practice Guidance on the clinical assessment and management of nonalcoholic fatty liver disease. Hepatology.

[92] European Association for the Study of the Liver, European Association for the Study of Diabetes, European Association for the Study of Obesity (2024). EASL-EASD-EASO Clinical Practice Guidelines on the management of metabolic dysfunction-associated steatotic liver disease (MASLD): Executive Summary. Diabetologia.

[93] California Department of Public Health (2022). Asian and Pacific Islander Data Disaggregation Highlights, California Assembly Bill 1726.

[94] Kauh T.J., Read J.G., Scheitler A.J. (2021). The Critical Role of Racial/Ethnic Data Disaggregation for Health Equity. Popul. Res. Policy Rev..

[95] Hu R., Ying X., Ng N., Lieu R., Jesudian A., Rosenblatt R., Silberstein P., Lucero C. (2024). Treatment and Survival Disparities in Asian Americans with Hepatocellular Carcinoma: The Need to Disaggregate a Diverse Cohort. J. Clin. Gastroenterol..

[96] Chen M.S., Lee R.J., Madan R.A., Ta Park V., Shinagawa S.M., Sun T., Gomez S.L. (2022). Charting a Path Towards Asian American Cancer Health Equity: A Way Forward. J. Natl. Cancer Inst..

[97] Pham C., Sin M.-K. (2021). Use of Electronic Health Records at Federally Qualified Health Centers: A Potent Tool to Increase Viral Hepatitis Screening and Address the Climbing Incidence of Liver Cancer. J. Cancer Educ..

[98] Zhao J., Han X., Nogueira L., Fedewa S.A., Jemal A., Halpern M.T., Yabroff K.R. (2022). Health insurance status and cancer stage at diagnosis and survival in the United States. CA Cancer J. Clin..

